# A systematic review of neuroimaging studies of clozapine-resistant schizophrenia

**DOI:** 10.1038/s41537-023-00392-7

**Published:** 2023-09-26

**Authors:** Tiffanie Sze Wing Pang, Johnny Siu Wah Chun, Ting Yat Wong, Sin Ting Chu, Chak Fai Ma, William G. Honer, Sherry Kit Wa Chan

**Affiliations:** 1 Department of Psychiatry, School of Clinical Medicine, Li Ka Shing Faculty of Medicine, The University of Hong Kong, Hong Kong SAR, China; 2 Department of Psychology, The Education University of Hong Kong, Hong Kong SAR, China; 3 School of Nursing, The Hong Kong Polytechnic University, Hong Kong SAR, China; 4 Department of Psychiatry, The University of British Columbia, Vancouver, Canada; 5 The State Key Laboratory of Brain and Cognitive Sciences, The University of Hong Kong, HKSAR, Hong Kong SAR, China

**Keywords:** Schizophrenia, Schizophrenia

## Abstract

This systematic review aimed to review neuroimaging studies comparing clozapine-resistant schizophrenia patients with clozapine-responding patients, and with first-line antipsychotic responding (FLR) patients. A total of 19 studies including 6 longitudinal studies were identified. Imaging techniques comprised computerized tomography (CT, *n* = 3), structural magnetic resonance imaging (MRI, *n* = 7), magnetic resonance spectroscopy (MRS, *n* = 5), functional MRI (*n* = 1), single-photon emission computerized tomography (SPECT, *n* = 3) and diffusion tensor imaging (DTI, *n* = 1). The most consistent finding was hypo-frontality in the clozapine-resistant group compared with the clozapine-responding group with possible differences in frontal-striatal-basal ganglia circuitry as well as the GABA level between the two treatment-resistant groups. Additional statistically significant findings were reported when comparing clozapine-resistant patients with the FLR group, including lower cortical thickness and brain volume of multiple brain regions as well as lower Glx/Cr level in the dorsolateral prefrontal cortex. Both treatment-resistant groups were found to have extensive differences in neurobiological features in comparison with the FLR group. Overall results suggested treatment-resistant schizophrenia is likely to be a neurobiological distinct type of the illness. Clozapine-resistant and clozapine-responding schizophrenia are likely to have both shared and distinct neurobiological features. However, conclusions from existing studies are limited, and future multi-center collaborative studies are required with a consensus clinical definition of patient samples, multimodal imaging tools, and longitudinal study designs.

## Introduction

The clinical course of schizophrenia shows huge interpatient variability, ranging from complete remission to persistent severe symptoms with significant functional impairment^1,2^. Despite adequate trials of at least 2 antipsychotic medications with sufficient dose and duration, ~15–30% of patients experience persistent symptoms^3–5^, and are defined as treatment-resistant schizophrenia (TRS)^6^. At present, clozapine is the only antipsychotic medication that is effective in ameliorating symptoms of TRS^7^ with evidence showing clinical efficacy in both short and long-term studies^8–11^. However, 30–70% of patients with TRS show inadequate response to clozapine^3,12^, and are categorized as ultra-treatment-resistant schizophrenia (UTRS)^6^. UTRS patients are associated with poorer clinical and functional outcomes compared to non-treatment-resistant schizophrenia (NTRS) and TRS patients who responded to clozapine^5^. Though delay in clozapine prescription is consistently identified as a factor associated with poor clozapine response^5,13^, multiple barriers to clozapine prescription are suggested including concerns of side effects^14^. Understanding factors associated with clozapine non-response, including neurobiological mechanisms may contribute to the development of targeted interventions to improve outcomes. Of the many studies examining predictors or associated factors contributing to clozapine responses, relatively few consistent effects were reported including younger age, fewer negative symptoms at onset and paranoid schizophrenia subtypes^15^; consistent biological predictors were lacking^16^.

Earlier studies suggested that a disturbance in dopaminergic transmission is central to schizophrenia^17^, with a growing focus on presynaptic dopaminergic dysfunction^18^. A meta-analysis of 44 studies using positron emission tomography (PET) or single-photon emission computed tomography (SPECT) reported a significant increase in synthesis and release of striatal dopamine in patients with schizophrenia^19^. Furthermore, the magnitude of dopamine synthesis increase predicted the clinical efficacy of dopamine receptor antagonist antipsychotics, and thus the treatment response^20,21^. In contrast, a cross-sectional study found a lower dopamine synthesis capacity in TRS than NTRS patients^22^. Coupled with the lower affinity of clozapine for dopamine receptors^23^, a different underlying of pathophysiology for TRS was proposed, as well as the possibility of using response to antipsychotic medications to characterize biologically distinct subtypes of illness^24^.

Evidenced by the two meta-analyses of ^1^H magnetic resonance spectroscopy (^1^H-MRS) studies, glutamate hypothesis is a complementary theory of schizophrenia, highlighting the role of upstream N-methyl-D-aspartate (NMDA) receptor hypofunction in causing a downstream cascade of neurotoxicity^25,26^. In more recent cross-sectional studies, TRS patients showed significantly higher glutamate levels in the anterior cingulate cortex (ACC) than the NTRS patients^27^ and healthy controls^28^. Moreover, higher ACC glutamate levels were associated with poorer treatment response to non-clozapine antipsychotics^29^. Meta-analyses of structural magnetic resonance imaging (MRI) studies reported subcortical volumetric reduction in schizophrenia patients compared to healthy subjects^30,31^. Specifically, cortical thinning was observed in the dorsolateral prefrontal cortex (DLPFC) of TRS patients compared to NTRS patients^32^. These neuroimaging studies highlighted the possibility of different biological mechanisms in TRS and NTRS and suggest reducing ACC glutamate levels may be a strategy for intervention in TRS.

Over decades, understanding the mechanisms of clozapine in TRS has been challenging due to the heterogeneity of TRS and the complex interaction between receptors and neurotransmitter systems^33^. Despite growing evidence supporting structural brain changes and differential neural mechanisms between NTRS and TRS, the neuroimaging evidence comparing TRS groups, including those who respond to clozapine (CRS) with UTRS is sparse and inconsistent^16^. An improved, systematically derived understanding of the neurobiological substrates of TRS as well as differences between CRS and UTRS would contribute to understanding the mechanisms of clinical efficacy of clozapine in TRS, and could facilitate greater application of existing clozapine treatment as well as facilitate development of novel interventions.

The aim of this review was to address the current research gap by systematically reviewing neuroimaging studies comparing patients with UTRS with other stages of the illness, particularly TRS responding to clozapine to provide insight on potential distinct neural mechanisms among UTRS patients. The results may provide further information on the possible presence of unique neurobiological characteristics of different subtypes of schizophrenia corresponding to characteristics of treatment response.

## Methods

### Literature review

Systematic searches of relevant articles were conducted from the electronic database, including Ovid Medline, Embase, Pubmed, PsychINFO, and Web of Science, from inception to 20 August 2022. An additional search was conducted from 21–30 August 2022 to confirm the inclusion of all relevant studies. Studies that were published in English in a peer-reviewed journal with study samples including schizophrenia, schizoaffective disorder, and schizophreniform disorders according to Diagnostic and Statistical Manual of Mental Health (DSM) or International Classification of Diseases (ICD) criteria, having an operationalized definition of UTRS status with comparison of CRS and patients with other treatment response characteristics using any form of neuroimaging approach were included. All cross-sectional and longitudinal studies were included. Conference abstracts, theses, and editorials were excluded. References from other review articles were examined for relevant studies. The review protocol was registered in the public domain (PROSPERO [International Prospective Register of Systematic Reviews] number: CRD42020203527).

**Fig. 1:**
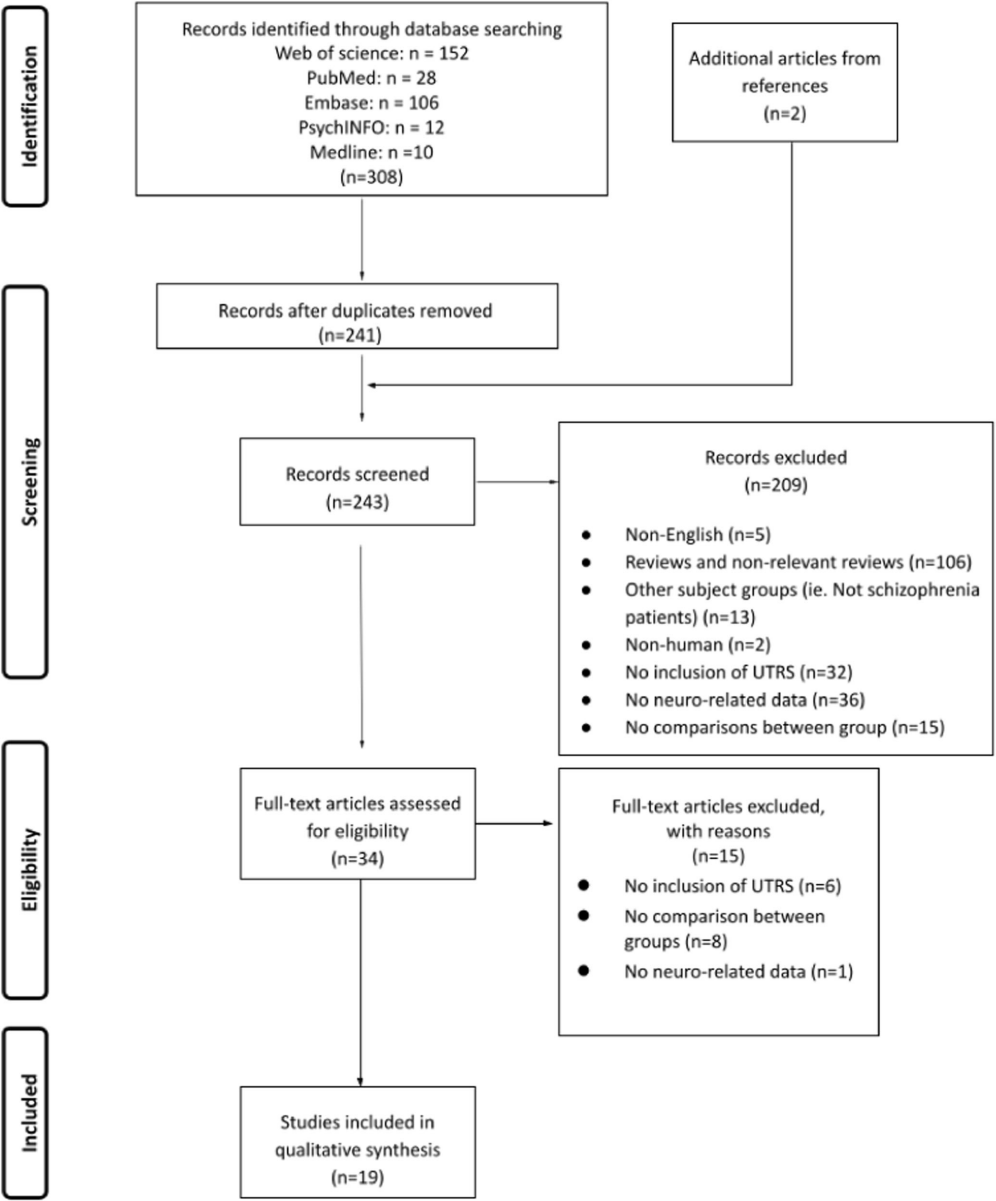
PRISMA flowchart. PRISMA flowchart of the systematic review process.

An electronic database search was conducted using the following syntax as search terms: (“ultra-treatment resistant”) OR (“ultra-resistant schizophrenia”) OR (“non clozapine responder schizophrenia”) OR (“clozapine resistant schizophrenia”) OR (“treatment refractory schizophrenia”) OR (“clozapine nonresponder”) AND (magnetic resonance imaging OR MRI OR functional magnetic resonance imaging spectroscopy OR fMRI OR magnetic resonance spectroscopy OR MRS OR voxel-based morphometry OR VBM OR positron emission tomography OR PET or diffusor tensor imaging OR DTI OR single-photon emission computed tomography OR SPECT OR computed tomography OR CT OR Diffusion-weighted magnetic resonance imaging OR DWI). The results of the systematic review are reported based on the PRISMA guidelines (Fig. 1).

### Data extraction

After removal of duplicates, one of the reviewers (TP) conducted a first-level screening of the titles and abstracts. References of included studies were screened for eligibility. Eligible studies were selected for full review and data extraction. A second reviewer (SKWC) independently conducted the review process. Inconsistencies were resolved through consensus meetings.

Basic information from the eligible studies was extracted independently by two reviewers, including study characteristics (author, publication year, study design, sample size, demographics of the target population, neuroimaging modality), participant details (number of subjects per group), clinical characteristics, statistical analysis used, and main study outcomes. Due to different definitions of UTRS, details of the inclusion criteria and definition of each patient group were documented. Inconsistencies were resolved through consensus meetings.

To align the different names and definitions of the illness status used in various studies, the following definitions are used in the current review: first-line responders (FLR) are those who show adequate response to non-clozapine antipsychotic medications; treatment-resistant schizophrenia (TRS) are those who failed to show an adequate response to at least 2 previous non-clozapine antipsychotics with at least 6 to 8-week trials; TRS that respond to clozapine (CRS) are TRS patients with symptomatic improvement after receiving adequate clozapine trials; TRS that do not respond to clozapine (UTRS) are TRS who failed to show an optimal response after adequate clozapine trials. Studies were grouped based according to brain region of interest and imaging modalities used.

### Study quality

Joanna Briggs Institute (JBI) appraisal tools were used to assess the methodological quality of all included studies. This tool allows consistent and transparent judgment of the quality of parameters including recruitment procedures, sample size, and the degree of appropriateness of statistical analysis. JBI critical appraisal tools for cohort and longitudinal studies were used as appropriate. These tools are comprised of 10 questions that address study design, the methodology, and the statistical analysis used and for each question, the risk of bias assessed using ‘Yes’, ‘No’, ‘Unclear’, and ‘Not applicable’. Two raters (T.P and D.M) completed the critical appraisal tool independently and any discrepancies were resolved through discussion.

## Results

**Fig. 2:**
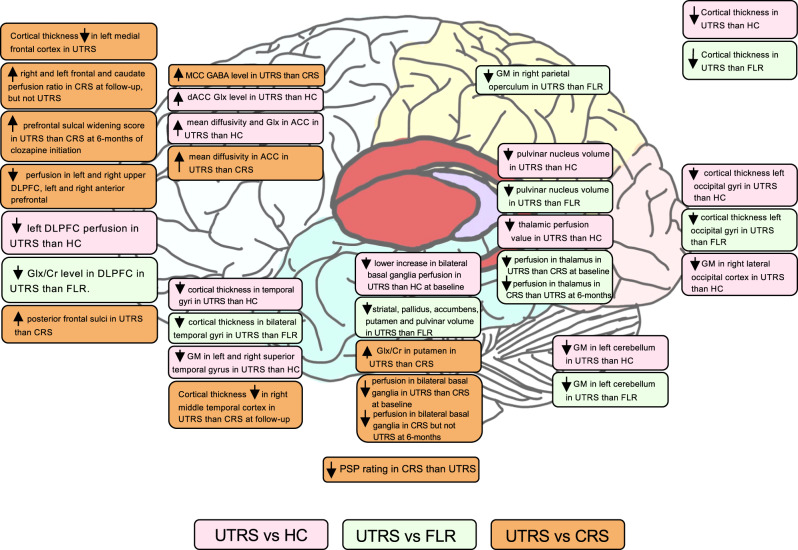
Summary figure for the result of the study. UTRS ultra-treatment-resistant schizophrenia, CRS clozapine responder schizophrenia, FLR first line responder schizophrenia, HC healthy controls, GM gray matter, PSP prefrontal sulcal prominence, Cr creatine, Glu glutamate, Glx glutamine+glutamate, DLPFC dorsolateral prefrontal cortex, ACC anterior cingulate cortex, dACC dorsal anterior cingulate cortex.

A total of 308 studies were identified based on search keywords; 19 studies fulfilled the inclusion criteria and were finally included in the current review (Fig. 1). Of the 19 studies, three used computerized tomography (CT), seven used structural magnetic resonance imaging (MRI), five used magnetic resonance spectroscopy (MRS), one reported functional MRI, three used single-photon emission computerized tomography (SPECT), and one used diffusion tensor imaging (DTI). Six were longitudinal and thirteen were cross-sectional studies (Table 1). All studies reported operational definitions of the UTRS and CRS while nine studies included FLR with clear operational definitions and the total sample size of each study ranging from 22 to 152 (Table 1). Study quality is reported in Supplementary Table 1 (for cross-sectional studies) and 2 (for longitudinal studies). A comprehensive overview of the significant findings is displayed in Fig. 2.

**Table 1:** Summary of the included study designs and characteristics.

Author	Year	Research design	Sample	Mean Clozapine dosage	Definition of treatment response			Neuroimaging modality
					First-line responder (FLR)	Clozapine responder (CRS)	Clozapine non-responder (UTRS)	
Ochi et al.	2022^42^	Cross-sectional	Total *N* = 152 21 UTRS (0 from Tokyo cohort); 38 CRS (23 Tokyo cohort, 15 Toronto cohort); 41 FLR (24 Tokyo cohort, 17 Toronto) 52 HC (26 Tokyo cohort, 26 Toronto cohort)	Not reported	(1) Responder to first-line antipsychotics	(1) History of failure of standard treatment with at least two previous non-clozapine antipsychotics (2) Subsequent successful treatment response to clozapine	(1) History of failed standard treatment with at least two previous non-clozapine antipsychotics (2) Did not respond to clozapine	Structural MRI 1H-MRS Diffusion MRI
Ueno et al.	2022^49^	Cross-sectional	Total *N* = 98: 22 UTRS; 25 CRS; 16 FLR: 35 HC	UTRS: 400.0 ± 198.4; CRS: 383.0 ± 165.2	(1) Current use of non-clozapine single antipsychotic (2) Successful treatment response	(1) Current monotherapy with clozapine (2) History of failure of standard treatment with at least two previous non-clozapine antipsychotics (3) Subsequent successful treatment response to clozapine	(1) Currently monotherapy with clozapine (2) History of failed standard treatment with at least two previous non-clozapine antipsychotics (3) Treatment failure with clozapine after taking for >=6 weeks at a minimum dosage of 300 mg/day	1H-MRS
Iwata et al.	2021^48^	Cross-sectional	Total = 98 24 UTRS; 27 CRS; 21 FLR; 26 HC	UTRS: 429.1 ± 124.3; CRS: 351.4 ± 134.5	(1) Current treatment of a single non-clozapine antipsychotic (2) Treatment response	(1) Current treatment of clozapine (2) History of treatment failure to optimal treatment with at least 2 previous non-clozapine antipsychotics (3) Subsequent successful treatment response to clozapine	(1) Current treatment of clozapine (2) History of treatment failure to optimal treatment with at least 2 previous non-clozapine antipsychotics (3) Subsequent treatment failure with clozapine after patients had taken clozapine for ≥6 weeks at a minimum dose of 300 mg/day	1H-MRS
Kim et al.	2020^51^	Cross-sectional	Total: 103 27 UTRS 20 CRS 21 FLR 26 HC	Not reported	(1) Intake of antipsychotic (other than clozapine) for ≥6 weeks with adequate response	(1) A history of suboptimal response to ≥2 antipsychotic trials other than clozapine (2) Intake of clozapine (CPZ equivalent dose of ≥400 mg/day) for ≥6 consecutive weeks (3) CGI-S ≤ 3 (4) All PANSS-positive items scored ≤3 (5) No symptomatic relapse in the past 3 months	(1) History of suboptimal response to ≥2 antipsychotic trials other than clozapine; (2) CPZ equivalent dose ≥400 mg for ≥6 consecutive weeks with suboptimal clinical response (3) CGI-S score ≥4 (4) 2 items on PANSS positive symptoms scored. ≥4	Structural MRI
McNabb et al.	2020^39^	Cross-sectional	Total: 70 14 UTRS 18 CRS 18 FLR 20 HC	Not reported	(1) Responding well to first-line atypical antipsychotic monotherapy (2) Improvement in positive symptoms according to standard practice and treatment guidelines for schizophrenia	(1) Failed at least two previous six-to-eight-week trials of atypical antipsychotics and received clozapine at the time of screening [need to meet the minimum required for TRIPP]	(1) Failed at least two previous six-to-eight-week trials of atypical antipsychotics and had also failed an adequate trial of clozapine monotherapy (at least 8 weeks post titration) [need to meet the minimum required for TRIPP] (2) Subsequent need for additional antipsychotics	DTI
Shah et al.	2020^41^	Cross-sectional	Total = 94 24 UTRS 25 CRS; 19 FLR; 26 HC	Not reported	(1) Intake non-clozapine antipsychotic with an adequate response	(1) Failed to respond to at least 2 non-clozapine antipsychotics (CPZ equivalent dose ≥400 mg/day) treatment, each administered ≥6 weeks (2) CGI-S score ≤3 (3) All positive symptoms items of PANSS scored ≤3 (4) No relapse during the past 3 months	(1) Intake of clozapine at the time of study; (2) Met the criteria for TRS (3) CGI-S score ≥4 (4) All positive symptoms items of PANSS scored ≥3 (5) Have relapse during past 3 months	Structural MRI, 1H-MRS
Tronchin et al.,	2020^50^	Longitudinal; 6 to 9 months pre and post-clozapine treatment	Total = 64; 33 TRS (16 CRS; 17 UTRS); 31 HC	349.2 ± 17.8	N/A	(1) Failed to respond to at least 2 antipsychotic medications, including at least 1 atypical antipsychotic drug, with a prolonged period of moderate to severe positive and/or negative symptoms (2) >50% reduction from baseline total PANSS score	(1) Failed to respond to at least 2 antipsychotic medications, including at least 1 atypical antipsychotic drug, with a prolonged period of moderate to severe positive and/or negative symptoms (2) ≤50% reduction from baseline total PANSS score	Structural MRI
Iwata et al.	2019^46^	Cross-sectional	Total = 100: 26 UTRS; 21 CRS; 26 HC	UTRS: 428.8 ± 119.5; CRS: 351.4 ± 134.5	(1) Current intake of non-clozapine single antipsychotic (2) Response to treatment	(1) Currently intake of clozapine (2) A history of failed treatment to ≥2 non-clozapine treatment (3) Subsequent response to clozapine after patients had taken clozapine (≥300 mg/day) for ≥6 weeks	(1) Current intake of clozapine; (2) A history of failed treatment to ≥2 previous non-clozapine treatment; (3) Failed to response to clozapine (≥300 mg/day) after taken for ≥6 weeks	1H-MRS
McNabb et al.	2018^40^	Cross-sectional	Total = 69 16 UTRS; 18 CRS; 18 FLR; 17 HC	Not reported	(1) Responding well to first-line atypical antipsychotic monotherapy (2) Improvement in positive symptoms according to standard practice and treatment guidelines for schizophrenia	(1) Failed at least two previous six-to-eight-week trials of atypical antipsychotics and received clozapine at the time of screening [need to meet the minimum required for TRIPP].	(1) Failed at least two previous six-to-eight-week trials of atypical antipsychotics and had also failed an adequate trial of clozapine monotherapy (at least 8 weeks post titration) [need to meet the minimum required for TRIPP]. (2) Subsequent need for additional antipsychotics	fMRI
Ahmed et al.	2015^38^	Longitudinal	Total = 64 33 TRS (20 CRS; 13 UTRS) 31 HC	UTRS: 383.3 ± 105.5; CRS: 321.3 ± 87.5	N/A	(1) >50% reduction from baseline total PANSS score	(1) ≤50% reduction from baseline total PANSS score	Structural MRI
Anderson et al.	2015^37^	Cross-sectional	Total *N* = 72 15 UTRS; 19 CRS; 18 FLR; 20 HC	Not reported	(1) Responding well to first-line atypical antipsychotic monotherapy (2) Improvement in positive symptoms according to standard practice and treatment guidelines for schizophrenia	(1) Failed at least two previous six-to-eight-week trials of atypical antipsychotics and received clozapine at the time of screening [need to meet the minimum required for TRIPP]	(1) Failed at least two previous six-to-eight-week trials of atypical antipsychotics and had also failed an adequate trial of clozapine monotherapy (at least 8 weeks post titration) [need to meet the minimum required for TRIPP]. (2) Subsequent need for additional antipsychotics	Structural MRI
Goldstein et al.	2015^47^	Cross-sectional	Total = 58: 11 UTRS; 16 CRS; 15 FLR; 16 HC	UTRS: 433.3 ± 154.1; CRS: 385.9 ± 181.0	(1) Responding well to first-line atypical antipsychotic monotherapy (2) Improvement in positive symptoms according to standard practice and treatment guidelines for schizophrenia	(1) Failed at least two previous six-to-eight-week trials of atypical antipsychotics and were receiving clozapine at the time of screening [need to meet the minimum required for TRIPP]	(1) Failed at least two previous six-to-eight-week trials of atypical antipsychotics and had also failed an adequate trial of clozapine monotherapy (at least 8 weeks post titration) [need to meet the minimum required for TRIPP] (2) Subsequent need for additional antipsychotics	1H-MRS
Ertugrul et al.	2009^45^	Longitudinal, 8 weeks pre and post clozapine treatment	Total = 22 11 UTRS; 11 CRS	Not reported	N/A	(1) Failed at least 2 previous 6-8 weeks trials of atypical antipsychotics (2) Received 8-weeks of clozapine (3) ≤25% of their PANSS total score compared to baseline	(1) Received 8-weeks of clozapine (2) ≥25% of their PANSS total score compared to baseline	1H-MRS, SPECT
Konicki et al.	2001^35^	Cross-sectional	Total = 65 10 UTRS; 26 CRS	UTRS: 493 ± 67; CRS: 490 ± 32	N/A	Clinical Global Impression (CGI) - change score ≥2 (much improved) at 6 months of clozapine treatment	Clinical Global Impression (CGI)-change score ≤4 (unchanged) at 6 months of clozapine treatment	CT scan
Scheepers et al.	2001^52^	Longitudinal, 24 weeks pre and post clozapine treatment	Total = 26 26 TRS; (13 UTRS, 13 CRS)	Not reported	N/A	(1) Treated with ≥1 typical antipsychotic for ≥4 week (2) Failed to show adequate responses to treatment with typical antipsychotics (3) ≤20% of their total PANSS score compared to the baseline	(1) Treated with at least 1 typical antipsychotic for a minimum of 4 weeks (2) Failed to show adequate responses to treatment with typical antipsychotics (3) ≥20% of their total PANSS score compared to baseline	Structural MRI
Rodríguez et al.	1997^44^	Longitudinal, 6 months pre and post clozapine treatment	Total = 67 22 UTRS; 17 CRS; 28 HC	Not reported	N/A	(1) Absence of response to adequate doses of 2 antipsychotic treatments (2) >50% decrease in global clinical scores (SAPS & SANS) (3) CGI score <3 (4) Currently intake of clozapine	(1) Absence of response to adequate doses of 2 antipsychotic treatments (2) <50% decrease in global clinical scores (SAPS & SANS) (3) CGI score >3 (4) Currently intake of clozapine	SPECT
Rodríguez et al.	1996^43^	Longitudinal, 6 months pre and post clozapine treatment	Total = 24 11 UTRS; 13 CRS	Not reported	N/A	(1) Absence of response to adequate doses of 2 antipsychotic treatments (2) >50% decrease in global clinical scores (SAPS & SANS) (3) CGI score <3 (4) Currently intake of clozapine	(1) Absence of response to adequate doses of 2 antipsychotic treatments (2) <50% decrease in global clinical scores (SAPS & SANS) (3) CGI score >3 (4) Currently intake of clozapine	SPECT
Honer et al.	1995^34^	Cross-sectional: at discharge	Total = 42 27 UTRS; 15 CRS	UTRS: 400 ± 200; CRS: 430 ± 190	N/A	(1) CGI Severity score at discharge ≤4 (moderately ill) (2) Improvement CGI score ≤2 (much improved)	(1) CGI Severity score at discharge ≥5 (markedly ill) (2) Improvement CGI score ≥3 (minimally improved)	CT scan
Friedman et al.	1991^36^	Cross-sectional: at 6 weeks	Total = 34 12 UTRS; 22 CRS	Not reported	N/A	(1) Improved from 15–40% using BPRS total after 6 weeks of clozapine initiation	(1) Patients who either worsened or improved by less than 15% using BPRS total	CT scan

*FLR* non-treatment-resistant schizophrenia, *TRS* treatment-resistant schizophrenia, *UTRS* ultra-treatment-resistant schizophrenia, *CRS* clozapine response schizophrenia, *PANSS* Positive and Negative Symptoms Scale, *CGI* Clinical Global Impression scale, *CGI-S* Clinical Global Impression Severity Scale, *SAPS* Scale for the Assessment of Positive Symptoms, *SANS* Scale for the Assessment of Negative Symptoms, *BPRS* Brief Psychiatric Rating Scale, *CPZ* chlorpromazine, *MRI* magnetic resonance imaging*, fMRI* functional magnetic resonance imaging, *1H-MRS* proton magnetic resonance spectroscopy, *SPECT* single-photon emission computed tomography, *DTI* diffusion tensor imaging, *N/A* not applicable.

### Global brain structural and connectivity

Five studies reported the global structural differences between UTRS patients and patients with other treatment response characteristics (Table 2). An early cross-sectional CT study demonstrated significantly increased global sulcal widening and significantly higher total cortical score in UTRS compared to CRS^34^. However, one CT study with 10 UTRS patients and 26 CRS reported no significant difference in general sulcal widening between the two groups^35^ and another CT scan study with 12 UTRS patients and 22 CRS also found no significant differences in ventricular brain ratio between the two groups^36^. Compared with healthy controls (HC) and FLR, patients with both treatment-resistant groups had significantly smaller overall gray matter volume (GM) and all patient groups had smaller whole brain volume and white matter (WM) volume than the healthy controls. However, no significant differences were seen between the UTRS and the CRS^37^. In a longitudinal study of response to clozapine, marginally more cortical thinning of the left medial frontal cortex and the right middle temporal cortex was seen for the UTRS in comparison with clozapine responders, but overall differences in brain volumes and cortical thickness between patients and healthy controls were reported at either time point^38^.

**Table 2:** Global structural differences and functional connectivity.

Author	Design	Modality	Analysis tool	Regions of Interest	Sample	Results		
						UTRS compared HC	UTRS compared FLR	UTRS compared CRS
McNabb et al., 2020^39^	Cross-sectional	DTI (3.0 T)	FSL	White Matter microstructure	14 UTRS; 18 CRS; 18 FLR; 20 HC	Not significant	Not significant	UTRS has a higher mean FA than CRS (*p* = 0.03, uncorrected) in the right superior longitudinal fasciculus (including the temporal component of the superior longitudinal fasciculus) but it did not survive correction for multiple testing.
McNabb et al., 2018^40^	Cross-sectional	fMRI (3.0 T)	NBS	Whole brain functional connectivity	16 UTRS; 18 CRS; 18 FLR; 17 HC	UTRS had weaker connectivity in 3 networks: cerebellar-frontal (*p* < 0.012, corrected), cingulo-frontal-temporal (*p* = 0.036, corrected) and fronto-parietal (*p* = 0.036, corrected)	Not significant	Not significant
Ahmed et al., 2015^38^	Longitudinal: 6-9 months pre- and post- clozapine	MRI (1.5 T)	FreeSurfer and SIENA	Cortical thinning, morphological brain changes (Whole brain)	33 TRS (13 UTRS; 20 CRS) 31 HC	N/A	N/A	UTRS had marginally greater cortical thinning of the left medial frontal cortex and the right middle temporal cortex compared with the CRS (*p* = 0.05). No correlation of clinical changes with cortical thinning.
Anderson et al., 2015^37^	Cross-sectional	MRI (3.0 T)	FSL-VBM	Global brain, GM, WM, ventricular cerebrospinal fluid volume, regional GM (whole brain)	15 UTRS; 19 CRS; 18 FLR; 20 HC	UTRS had significantly smaller whole brain volume (*p* < 0.001), GM volume (*p* < 0.001), WM volume (*p* = 0.007), peripheral cortex GM volume (*p*, 0.001) and larger ventricular CSF volume (*p,* 0.007).	UTRS had smaller whole brain volume (*p* = 0.002), GM volume (*p* < 0.001), peripheral cortex GM volume (*p* < 0.001) and larger ventricular CSF volume (*p* = 0.019).	No significant differences in mean brain volumes, GM volume, WM volume, peripheral cortex GM volume nor in ventricular CSF volume.
Konicki et al., 2001^35^	Cross sectional	CT	Sulcal widening index	general sulcal widening	10 UTRS; 26 CRS	N/A	N/A	No significant difference of general sulcal widening score
Honer et al., 1995^34^	Cross-sectional: at discharge	CT	Sum of sulcal width scores for each of 5 different brain regions	Cortical and ventricular regions	27 UTRS; 15 CRS	N/A	N/A	UTRS global sulcal widening was greater than CRS (*p* = 0.01) and the total cortical score in UTRS was significantly higher than CRS (*p* < 0.05). No significant differences in ventricular score were found.
Friedman et al., 1991^36^	Cross sectional	CT	Ventricular brain ratio (VBR) by compensating polar planimeter	whole brain and ventricles	12 UTRS; 22 CRS	N/A	N/A	No significant difference was found in VBR between groups.

*UTRS* ultra-treatment-resistant schizophrenia, *CRS* clozapine responder schizophrenia, *TRS* treatment-resistant schizophrenia, *FLR* first line responder schizophrenia, *HC* healthy controls, *MRI* magnetic resonance imaging, *CT* computerized tomography, *SPECT* Single-photon emission computed tomography, *1H-MRS* Proton magnetic resonance spectroscopy, *PET* positron emission tomography, *DTI* Diffusion tensor imaging, *FSL* FMRIB Software Library, *VBM* Voxel-Based Morphometry, *FA* fractional anisotropy, *GM* gray matter, *WM* white matter, *CSF* Cerebrospinal fluid, *N/A* not applicable.

Only one study reported the white matter microstructure using track-based spatial analysis of diffusion tensor imaging data, and a significantly higher fractional anisotropy (FA) of the right superior longitudinal fasciculus in the UTRS patients compared with the CRS patients was found^39^. However, the significance disappeared after adding head motion as a regressor. Furthermore, CRS patients had overall lower FA than health controls, the FLR, and the UTRS patients, though none of the post-hoc analyses survived corrections for multiple testing^39^. Only one functional MRI study investigated whole brain functional connectivity and reported that UTRS had the weaker connectivity in 3 networks including the cerebella-frontal, cingulo-frontal-temporal, and fronto-parietal when compared with healthy controls but no differences when compared to other patient groups^40^. The connectivity between controls, FLR, and CRS patients was found to be similar.

### Structural and functional differences based on brain regions

#### Frontal lobes

Thirteen studies reported results of analyses of frontal lobes; three used CT, three used MRI, three used SPECT, and four used MRS (Table 3). A larger frontal sulcal widening in UTRS compared with CRS was consistently reported in CT scan studies^34–36^. These findings suggest that enlarged frontal sulcal widening could be a unique structural characteristic of UTRS. UTRS showed significantly greater cortical thinning in the left medial frontal cortex than CRS longitudinally^38^, while no differences were seen when investigated cross-sectionally^41^. Another MRI study reported no differences in cortical thickness between UTRS and CRS but a significantly greater mean diffusivity was found in UTRS compared to CRS after controlling for age and gender^42^. However, a greater cortical thinning in the frontal regions was observed in UTRS compared with healthy controls and FLR regardless of age, sex, chlorpromazine equivalent (CPZ) daily dose, and PANSS total scores^41^. In addition, UTRS demonstrated a significantly greater mean diffusivity in the ACC compared to healthy controls after controlling for age and gender^42^.

**Table 3:** Magnetic resonance spectroscopy, structural, functional connectivity differences of frontal lobes and cingulate cortex between UTRS, CRS, FLR, and healthy controls.

Author	Design	Modality	Analysis tool	Sample	Results		
					UTRS compared HC	UTRS compared FLR	UTRS compared CRS
Ochi et al., 2022^42^	Cross-sectional	MRI and 1H-MRS (3.0 T)	OPNMF LC Model	21UTRS; 15 CRS; 17 FLR; 26 HC	UTRS had a reduced cortical thickness than HC *(p* < 0.001) and greater mean diffusivity in ACC *(p* = 0.039; *p* = 0.018*)*, particularly surface of anterior cingulate sulcus, after controlling for age and gender. Elevated Glx in ACC in UTRS than HC (*p* = 0.038)	No significant differences	UTRS had a greater mean diffusivity in ACC *(p* = 0.039; *p* = 0.018) than CRS after controlling for age and gender, particularly surface of anterior cingulate sulcus, but no differences were found in cortical thickness.
Ueno et al., 2022^49^	Cross-sectional	1H-MRS (3.0 T)	Gannet (version 2.3)	22 UTRS; 25 CRS; 16 FLR; 35 HC	No significant differences	No significant differences	UTRS had a significantly higher MCC GABA level than CRS (*p* = 0.01) after controlling for smoking status and antipsychotic dosage. No significant difference of Glx level between groups. No significant relationship between GABA and clinical or cognitive scores
Iwata et al., 2021^48^	Cross-sectional	1H-MRS (3.0 T)	LC Model	24 UTRS; 27 CRS; 21 FLR; 26 HC	No significant differences of GSH levels in the dACC among the groups.	No significant differences of GSH levels in the dACC among the groups.	No significant differences of GSH levels in the dACC among the groups.
Shah et al., 2020^41^	Cross-sectional	MRI (3.0 T)	RMINC statistical tool	24 UTRS; 25 CRS; 19 FLR; 26 HC	UTRS showed greater cortical thinning in frontal areas. [anterior cingulate and paracingulate gyrus (*left: p* = 0.009); bilateral supplementary motor area (*left and right: p* < 0.0005); superior frontal gyrus - medial (*left: p* = 0.001; *right: p* = 0.009); superior frontal gyrus - dorsolateral (*left and right: p* = 0.002*)*; middle frontal gyrus (*left: p* = 0.004*; right: p* < 0.0005)].	UTRS showed greater cortical thinning in the bilateral frontal gyri, left precentral gyrus, bilateral supplementary motor area, and left anterior cingulate gyrus.	No significant differences in cortical thinning between UTRS and CRS.
Iwata et al., 2019^46^	Cross-sectional	1H-MRS (3.0 T)	LC Model	26 UTRS; 27 CRS; 21 FLR; 26 HC	UTRS has a higher dACC Glx level than HC (*p* = 0.034) after controlling for age and GM ratio.	No significant differences	No significant differences
Ahmed et al., 2015^38^	Longitudinal: 6–9 months pre- and post- clozapine	MRI (1.5 T)	FreeSurfer and SIENA	33 TRS (20 CRS; 13 UTRS); 31 HC	N/A	N/A	UTRS marginally showed greater cortical thinning over time in the left medial frontal cortex (*p* = 0.05) but no volumetric differences were found between them.
Goldstein et al., 2015^47^	Cross-sectional	1H-MRS (3.0 T)	LC Model	11 UTRS; 16 CRS; 15 FLR; 16 HC	N/A	UTRS had a lower level of Glx/Cr in DLPFC (*p* = 0.04) compared to FLR even after CPZE was added as a covariate but no group differences were detected for NAA/Cr, Glu/Cr, or Cho/Cr.	No significant metabolites were detected in DLPFC.
Ertugrul et al., 2009^45^	Longitudinal: 8 weeks pre- and post- clozapine treatment	SPECT Radiotracer: Tc-99m HMPAO	Automatically defined ROIs were assigned to anatomical	11 UTRS; 11 CRS	N/A	N/A	A significant increase in right and left frontal (superior and medial)/caudate perfusion ratio (*p* = 0.007*; p* = 0.013) and in right and left frontal/caudate (*p* = 0.018*; p* = 0.029) were found in CRS but no significant changes were found in UTRS. CRS showed an increase in percentage change in perfusion ratio compared to baseline in left and right (superior and medial)/caudate perfusion ratio (*p* = 0.017; *p* = 0.004 respectively) when compared to UTRS.
Konicki et al., 2001^35^	Cross-sectional	CT	Sulcal widening index	10 UTRS; 26 CRS	N/A	N/A	UTRS had a significantly higher prefrontal sulcal widening score than CRS (*p* < 0.02) at 6-months of clozapine initiation
Rodríguez et al., 1997^44^	Longitudinal: 6 months pre- and post- clozapine treatment	SPECT Radiotracer: Tc-99m HMPAO	Automatically defined ROIs were assigned to anatomical regions by means of study of an anatomical atlas and PET/MRI studies	22 UTRS; 17 CRS; 28 HC	UTRS has a significantly lower left DLPFC perfusion value (*p <*0.001) but no significant differences were found in right upper dorsolateral perfusion compared to HC at baseline.	N/A	UTRS showed no significant perfusion changes whereas CRS showed a clear perfusion decrease in the left upper (*p* = 0.019) and right upper (*p* = 0.025) DLPFC and left (*p* = 0.007) and right (*p* = 0.015) anterior prefrontal.
Rodríguez et al., 1996^43^	Longitudinal: 6 months pre- and post- clozapine treatment	SPECT Radiotracer: Tc-99m HMPAO	The anatomical correspondences of the regions of interest were identified with a neuroanatomical atlas	11 UTRS; 13 CRS	N/A	N/A	No prefrontal changes in perfusion after clozapine treatment were observed in both UTRS and CRS.
Honer et al., 1995^34^	Cross-sectional	CT	Sum of sulcal width scores for each of 5 different brain regions	27 UTRS; 15 CRS	N/A	N/A	UTRS had larger posterior frontal sulci than CRS
Friedman et al., 1991^36^	Cross-sectional	CT	Prefrontal sulcal prominence (PSP) index	12 UTRS; 22 CRS	N/A	N/A	A significant difference of PSP was found between groups (*p* = 0.004)

*OPNMF* orthogonal projective non-negative matrix factorization, *UTRS* ultra-treatment-resistant schizophrenia, *CRS* clozapine responder schizophrenia, *TRS* treatment-resistant schizophrenia, *FLR* first line responder schizophrenia, *HC* healthy controls, *MRI* magnetic resonance imaging, *CT* computerized tomography, *SPECT* Single-photon emission computed tomography, *1H-MRS* Proton magnetic resonance spectroscopy, *PET* positron emission tomography, *DTI* Diffusion tensor imaging, *ROIs* regions of interest, *rCBF* regional cerebral blood flow, *GM* gray matter, *WM* white matter, *PSP* prefrontal sulcal prominence, *Cre* creatine plus phosphocreatine, *Cr* creatine, *NAA* N-acetyl aspartate, *Cho* choline-containing compounds, *Glu* glutamate, *Glx* glutamine+glutamate, *DLPFC* dorsolateral prefrontal cortex, *CPZE* chlorpromazine equivalents, *ACC* anterior cingulate cortex, *GSH* glutathione, *dACC* dorsal anterior cingulate cortex, *N/A* not applicable.

Three ^99^Tc-labeled hexamethyl-propylene-aminoxine (HMPAO) SPECT studies were conducted (Table 3). In the earliest study, neither UTRS nor CRS revealed any prefrontal perfusion changes at follow-up^43^. Shortly after, another study discovered a lower perfusion in the lower and right upper dorsal lateral prefrontal cortex (DLPFC) in UTRS compared to CRS at baseline. Furthermore, no changes in cortical frontal perfusion were observed in the UTRS group, whereas the responder group had a significant reduction in cortical frontal perfusion^44^. The later SPECT study found a significant increase in frontal/caudate perfusion ratio in the clozapine responder group but not in the non-responder group^45^. When compared with HC, a significantly lower perfusion value at the left DLPFC was noted in UTRS at baseline^44^. Furthermore, improvement of digit span-forward was significantly correlated with increase in percentage change in the right frontal/caudate perfusion ratio, whereas a significant relationship between improvement of word fluency and increase in percentage change in both right and left frontal /caudate perfusion ratio was seen.

Three spectroscopy studies found no group differences (clozapine responders or non-responders) in glutamate or Glx (glutamate and glutamine) levels in the dorsolateral prefrontal cortex (DLPFC)^46^, nor Glx/creatinine (Glx/Cr) level^47^, likewise for the glutathione level in the dorsal anterior cingulate cortex (dACC)^48^ (Table 3). One study reported higher Glx levels in the anterior cingulate cortex in the UTRS group compared with clozapine responders (Ochi et al., 2022). Another study reported that UTRS had higher Glx/Cr levels in the DLPFC compared with the FLR^47^. A recent study found a higher gamma-aminobutyric acid (GABA) level in mid-cingulate cortex (MCC) in UTRS compared with CRS after controlling for smoking status, sex, education, GM/(GM + WM), and age but no differences in Glx levels were found^49^. Furthermore, all four spectroscopy studies reported no significant relationship between the levels of these neuro-metabolites and clinical or cognitive function scores.

#### Parietal lobes

Two studies (one MRI and one SPECT) examined the parietal lobes (Table 4) without finding a difference in GM and WM volume nor in the perfusion changes between UTRS and CRS^37,45^. When compared with FLR, an extensive reduction of GM volume in the right parietal operculum was observed among UTRS^37^.

**Table 4:** Structural and functional differences between UTRS, CRS, FLR, and healthy controls of brain regions other than the frontal lobes and cingulate cortex.

Author	Design	Modality	Analysis tool	Sample	Results		
					UTRS compared HC	UTRS compared FLR	UTRS compared CRS
*Parietal lobes*							
Anderson et al., 2015^37^	Cross-sectional	MRI (3.0 T)	FSL-VBM	15 UTRS; 19 CRS; 18 FLR; 20 HC	N/A	UTRS showed less GM in the right parietal operculum (*p* = 0.008).	No significant differences in mean brain volumes.
Ertugrul et al., 2009^45^	Longitudinal: 8 weeks pre- and post- clozapine treatment	SPECT Radiotracer: Tc-99m HMPAO	Automatically defined ROIs were assigned to anatomical	11 UTRS; 11 CRS	No significant differences observed.	N/A	No significant differences observed.
*Occipital lobe*							
Shah et al., 2020^41^	Cross-sectional	MRI (3.0 T)	RMINC statistical tool	24 UTRS; 25 CRS; 19 FLR; 26 HC	A greater cortical thinning was found in UTRS compared to HC in the left occipital gyri (*p* < 0.05).	UTRS showed greater cortical thinning in the left occipital gyri (*p* < 0.05).	No significant differences
Anderson et al., 2015^37^	Cross-sectional	MRI (3.0 T)	FSL-VBM	15 UTRS; 19 CRS; 18 FLR; 20 HC	An extensive bilateral pattern of decreased GM in UTRS in the right lateral occipital cortex (*p* = 0.018).	No significant differences.	No significant differences.
Rodríguez et al., 1996^43^	Longitudinal: 6 months pre- and post- clozapine treatment	SPECT Radiotracer: Tc-99m HMPAO	The anatomical correspondences of the regions of interest were identified with a neuroanatomical atlas	11 UTRS; 13 CRS	N/A	N/A	No significant differences in the occipital lobe.
*Temporal lobe*							
Shah et al., 2020^41^	Cross-sectional	MRI (3.0 T)	RMINC statistical tool	24 UTRS; 25 CRS; 19 FLR; 26 HC	A greater cortical thinning was found in UTRS compared to HC in the temporal gyri (*p* < 0.05).	UTRS showed greater cortical thinning in the bilateral temporal gyri (*p* < 0.05).	No significant differences
Tronchin et al., 2020^50^	Longitudinal: 6-9 months pre- and post- clozapine	MRI (1.5 T)	FreeSurfer	33 TRS (16 CRS; 17 UTRS); 31 HC	N/A	N/A	No significant baseline differences between UTRS and CRS in lateral ventricles, hippocampus, and amygdala were found.
Ahmed et al., 2015^38^	Longitudinal: 6-9 months pre- and post- clozapine	MRI (1.5 T)	FreeSurfer and SIENA	33 TRS (20 CRS; 13 UTRS); 31 HC	N/A	N/A	At follow-up, UTRS marginally showed greater cortical thinning in the right middle temporal cortex *(p* = 0.05) but no volumetric differences were found between them.
Anderson et al., 2015^37^	Cross-sectional	MRI (3.0 T)	FSL-VBM	15 UTRS; 19 CRS; 18 FLR; 20 HC	An extensive bilateral pattern of decreased GM in UTRS in the right superior temporal gyrus (*p* = 0.002), left superior temporal gyrus (*p* < 0.001).	No significant differences.	No significant differences.
Rodríguez et al., 1996^43^	Longitudinal: 6 months pre- and post- clozapine treatment	SPECT Radiotracer: Tc-99m HMPAO	The anatomical correspondences of the regions of interest were identified with a neuroanatomical atlas	11 UTRS; 13 CRS	N/A	N/A	No significant differences in the anterior and posterior temporal lobes.
*Basal ganglia*							
Kim et al., 2020^51^	Cross-sectional	MRI (3.0 T)	BEaST	27 UTRS; 20 CRS; 21 FLR; 26 HC	N/A	A smaller mean striatal volume *(p* < 0.001), a smaller globus pallidus volume *(p* = 0.045), nucleus accumbens *(p* = 0.002), pre- and post-commissural putamen volume (*p* = 0.002*; p* < 0.001), and pulvinar nucleus volume *(p* = 0.012) were found in UTRS compared to FLR.	No significant differences in the striatum, globus pallidus, nucleus accumbens and pre- and post-commissural putamen.
Tronchin et al., 2020^50^	Longitudinal: 6-9 months pre- and post- clozapine	MRI (1.5 T)	FreeSurfer	33 TRS (16 CRS; 17 UTRS); 31 HC	N/A	N/A	No significant baseline volumetric differences were found in the caudate, putamen, pallidus and nucleus accumbens.
Goldstein et al., 2015^47^	Cross-sectional	1H-MRS (3.0 T)	LC Model	11 UTRS; 16 CRS; 15 FLR; 16 HC	No significant differences in metabolites in putamen were detected.	No significant differences in metabolites in putamen were detected.	CRS had a higher Glx/Cr than UTRS in putamen even after controlling for CPZE as a covariate *(p* = 0.02) but no other significant metabolites were detected in putamen.
Scheepers et al., 2001^52^	Longitudinal: 24 weeks pre- and post- clozapine	MRI (1.5 T)	Caudate volume measured manually using ANALYZE	26 TRS (13 UTRS, 13 CRS)	N/A	N/A	No significant differences in caudate volume
Rodríguez et al., 1997^44^	Longitudinal: 6 months pre- and post- clozapine treatment	SPECT Radiotracer: Tc-99m HMPAO	Automatically defined ROIs were assigned to anatomical regions by means of study of an anatomical atlas and PET/MRI studies	22 UTRS; 17 CRS; 28 HC	UTRS has a significantly lower increase in right basal ganglia (*p* < 0.001) and left basal ganglia *(p* < 0.01) perfusion compared to HC at baseline.	N/A	CRS showed a significant perfusion reduction in right *(p* = 0.005) & left *(p* = 0.001) basal ganglia but no significant perfusion changes in UTRS at 6-months. UTRS showed a lower perfusion in left *(p* < 0.001) and right *(p* = 0.002) basal ganglia than CRS at baseline.
Rodríguez et al., 1996^43^	Longitudinal: 6 months pre- and post- clozapine treatment	SPECT Radiotracer: Tc-99m HMPAO	The anatomical correspondences of the regions of interest were identified with a neuroanatomical atlas	11 UTRS; 13 CRS	N/A	N/A	Only CRS showed a decrease in perfusion after clozapine treatment in the left basal ganglia.
*Thalamus*							
Kim et al., 2020^51^	Cross-sectional	MRI (3.0 T)	BEaST	27 UTRS; 20 CRS; 21 FLR; 26 HC	A smaller pulvinar nucleus volume *(p =* 0.010*)* were found in UTRS compared to HC after controlling for age, sex, TBV, education, tobacco use, lifetime history of substance dependence or abuse.	A smaller pulvinar nucleus volume (*p* = 0.012) was found in UTRS compared to FLR after controlling for age, sex, TBV, education, tobacco use, lifetime history of substance dependence or abuse.	No significant volumetric differences in thalamus.
Tronchin et al., 2020^50^	Longitudinal: 6-9 months pre- and post- clozapine (1.5 T)	MRI	FreeSurfer	33 TRS (16 CRS; 17 UTRS); 31 HC	N/A	N/A	No significant baseline volumetric differences were found in the thalamus.
Rodríguez et al., 1997^44^	Longitudinal: 6 months pre- and post- clozapine treatment	SPECT Radiotracer: Tc-99m HMPAO	Automatically defined ROIs were assigned to anatomical regions by means of study of an anatomical atlas and PET/MRI studies	22 UTRS; 17 CRS; 28 HC	UTRS has a significantly lower thalamic perfusion value *(p* < 0.001) compared to HC at baseline.	N/A	UTRS showed no perfusion changes in the thalamus whereas CRS showed a significant perfusion decrease in the thalamus *(p* = 0.009) at 6 months. UTRS showed a lower perfusion in thalamus *(p* < 0.001) than CRS at baseline.
Rodríguez et al., 1996^43^	Longitudinal: 6 months pre- and post- clozapine treatment	SPECT Radiotracer: Tc-99m HMPAO	The anatomical correspondences of the regions of interest were identified with a neuroanatomical atlas	11 UTRS; 13 CRS	N/A	N/A	Only CRS showed a decrease in perfusion after clozapine treatment in the thalamus.
*Cerebellum*							
Anderson et al., 2015^37^	Cross-sectional	MRI (3.0 T)	FSL-VBM	15 UTRS; 19 CRS; 18 FLR; 20 HC	A decreased GM in UTRS, left cerebellum (MNI peak co-ordinate: -32, -60, -48; -32, -50, -24) *(p* = 0.010*; p* = 0.027).	UTRS showed less GM in the left cerebellum *(p* = 0.016).	No significant differences.

*UTRS* clozapine non-responder schizophrenia, *CRS* clozapine responder schizophrenia, *TRS* treatment-resistant schizophrenia, *FLR* first line responder schizophrenia, *HC* healthy controls, *MRI* magnetic resonance imaging, *SPECT* Single-photon emission computed tomography, *1H-MRS* Proton magnetic resonance spectroscopy, *PET* positron emission tomography, *ROIs* regions of interest, *BEaST* Brain Extraction based on nonlocal Segmentation Technique, *Cr* creatine, *NAA* N-acetyl aspartate, *Cho* choline-containing compounds, *Glu* glutamate, *Glx* glutamine+glutamate, *DLPFC* dorsolateral prefrontal cortex, *CPZE* chlorpromazine equivalents, *TBV* total brain volume, *MNI* Montreal Neurological Institue, *N/A* not applicable.

#### Occipital lobes

Three studies (two MRI and one SPECT) investigated the occipital lobes region (Table 4) reporting no significant difference in GM, WM, and CSF between UTRS and CRS^37,41^, and no significant difference in perfusion ratio between the groups^43^. However, UTRS showed a significant GM reduction in the right lateral occipital cortex when compared with healthy controls, whereas the CRS patients showed a significant GM reduction in the lateral occipital cortex when compared with the FLR group^37^. Although no significant differences in cortical thinning in occipital lobes were found between UTRS and CRS, a significantly greater cortical thinning in UTRS was found in occipital gyri when compared with healthy controls, in addition to a more extensive thinning in the left occipital gyri than FLR^41^.

#### Temporal lobes

Five studies examined temporal lobe regions (Table 4) with only one study reporting significant cortical thinning of the right middle temporal cortex in UTRS compared with CRS^38^. Other studies did not find any difference in cortical thickness^41^, no volumetric differences in lateral ventricle, hippocampus, and amygdala^50^, no GM volume differences of temporal cortex^37^, nor cerebral perfusion differences at the anterior and posterior temporal lobe^43^ between the two groups. When compared with FLR and HC, significant cortical thinning was found in UTRS^41^. A bilateral pattern of decreased GM volume was also found in the superior and middle temporal gyri when compared between UTRS and healthy controls^37^. Compared with FLR, a significant reduction of the GM volume in superior, middle, and inferior temporal gyri was seen in the CRS group^37^.

#### Basal ganglia

Six studies examined the basal ganglia region; three used MRI, one MRS, and two SPECT techniques (Table 4). All three MRI studies reported no significant volumetric differences in the basal ganglia region between UTRS and CRS in both a cross-sectional study^51^ and longitudinal studies^50,52^. However, UTRS showed a smaller mean striatal volume, globus pallidus, nucleus accumbens, pre- and post-commissural putamen, and pulvinar nucleus volume when compared against FLR^51^. Furthermore, both patient groups were found to have a reduction in volume of putamen and hippocampus compared with healthy controls^50^ but no differences were shown between UTRS and HC^51^.

The study examining glutamatergic function reported a significantly higher Glx/Cr in CRS than UTRS in putamen after controlling for CPZ but no other significant differences in metabolites were detected in putamen between UTRS and other comparison groups^47^. Lastly, one ^99^Tc-labeled HMPAO SPECT study^44^ noted decreased perfusion in the bilateral basal ganglia in UTRS compared to CRS, while the other indicated a decreased perfusion in the left basal ganglia in CRS instead^43^. When compared with HC, UTRS had significantly lower perfusion in the basal ganglia^44^. Furthermore, another ^99^Tc-labeled HMPAO SPECT study found a significant increase in right and left frontal/caudate perfusion ratio in the CRS compared with the UTRS^45^.

#### Thalamus

Four studies examined the thalamus region, two using MRI and two SPECT (Table 4). No significant volumetric differences in the thalamus between UTRS and CRS were observed in a cross-sectional^51^ or a longitudinal study^50^. A smaller pulvinar nucleus volume in UTRS compared to FLR and HC and a smaller mean thalamus volume was found between UTRS and HC after controlling for age, sex, total brain volume, education, tobacco use, life history of substance dependence or abuse^51^. In the two ^99^Tc-labeled HMPAO SPECT studies, both showed CRS had a significant decrease of perfusion in thalamus^43,44^.

#### Cerebellum

Only one MRI study examined the cerebellum region (Table 4), reporting no significant GM differences observed between the two treatment-resistant patient groups. A significant reduction of GM volume in the left cerebellum was found comparing the UTRS and the FLR patients as well as UTRS and the healthy controls^37^.

## Discussion

Certain neurobiological features of treatment-resistant schizophrenia appear categorically different from treatment-responsive patients and could be considered biomarkers^53^. Response to antipsychotic medications is further suggested as a subtyping strategy for patients with schizophrenia^54^. Despite the plethora of literature on biomarkers in predicting treatment-resistant schizophrenia as well as clozapine response^16^, there were only 19 neuroimaging studies identified in the current review specifically comparing UTRS patients with patients with other characteristics of treatment responsiveness, in particular clozapine response. Among these studies, comparisons of frontal lobe properties were reported most frequently (13 studies) and generated the greatest number of differences between groups. Four out of five studies reported a significant difference between UTRS and CRS, including lowered cortical thickness and widening of sulci, suggesting a general reduction of the frontal lobe volume in UTRS patients in comparison with patients who responded to clozapine. Furthermore, two SPECT studies both reported a significant reduction in perfusion of the frontal region and the frontal/caudate perfusion ratio in UTRS patients. One MRI study reported UTRS had a significantly greater mean diffusivity in ACC than CRS. Studies of other brain regions are much fewer, and all reported no differences in brain volumes. Only one MRS study reported a significantly lower Glx/Cr level in putamen in UTRS compared with CRS, one MRS study found a significantly higher GABA level in mid-cingulate cortex (MCC) in UTRS compared with CRS, and two SPECT study reported lower perfusion and lack of perfusion changes with clozapine treatment in the thalamus among the UTRS patients compared with the CRS patients. These findings suggested that the most pervasive and significant neurobiological differences between the UTRS patients compared with patients who responded to clozapine are likely to be in the frontal region. In fact, this is also reflected by the largely negative findings of global brain volume and cortical thickness comparison between the two treatment-resistant group patients. Furthermore, compared with healthy controls, the UTRS group was found to have lower brain functional connectivity of three networks, all involving the frontal region. But no difference in the CRS patients was found in comparison with controls. These findings align with previous reports on the presence of low prefrontal cortex activities in clozapine-resistant patients^16^ and relationship of hypo-frontality and clozapine treatment in an earlier systematic review^55^. Furthermore, an earlier systematic review identified 5 studies comparing clozapine responders and clozapine non-responders also similarly found involvement of the frontal region^53^.

Studies of other regions comparing UTRS and CRS patients are relatively few, and mostly with negative findings apart from basal ganglia and thalamus. One SPECT study reported both reduction in perfusion of the bilateral basal ganglia and thalamus in UTRS patients compared with the clozapine response group and two studies reported a lack of perfusion changes in the UTRS with clozapine initiation. Another SPECT study also suggested significantly lowered frontal/caudate perfusion ratio in the UTRS patients. Furthermore, one MRS study reported significantly higher Glx/Cr in CRS than UTRS in putamen. Previous review also reported the caudate volume and basal ganglia perfusion were related to clinical response to clozapine without specific comparison of UTRS and CRS^53^. Though all the positive findings were reported in only a single study, coupled with the findings of the frontal lobe region, it is possible that frontal-striatal-basal ganglia circuitry function may represent a distinct neurobiological marker of UTRS. However, further exploratory studies are required.

Only one DTI study compared the white matter microstructure of the two treatment-resistant groups. Surprisingly the CRS patients were found to have the lowest FA compared with all other groups (HC, FLP and UTRS). In particular, they had a significantly lower FA of the right superior longitudinal fasciculus compared with the UTRS. Though it was no longer significant after correction for multiple testing and adding the head motion as regressor, the preliminary results of this single study may indicate the possible presence of a unique pattern of white matter microstructure of patients who respond to clozapine. Lower FA indicates lower homogeneity of white matter tractography and poorer white matter integrity. However, a study of Williams syndrome found higher FA of the superior longitudinal fasciculus tract associated with poorer visual-spatial functioning^56^. A counterintuitive result of an early brain imaging study also found brain dysmorphology is related to better symptom improvement with clozapine^57^. These results place more complexity into the relationship between white matter microstructure and the clozapine responses of TRS patients.

There were only three MRS studies comparing the two treatment-resistant patient groups and only one study of GABA, that reported significantly higher GABA level in mid-cingulate cortex (MCC) in UTRS compared with CRS. A unique relationship between clozapine and GABA_B_ receptor-mediated inhibitory neurotransmission was reported^58^ as well as the binding properties of clozapine with the GABA_B_ receptor^59^. Therefore, it is possible that the GABA level may be a biomarker of clozapine-resistant TRS patients. However, replication studies are required. Furthermore, given that the MCC was the only region examined, studies of GABA level in other brain regions particularly in the frontal lobes are needed to further our understanding. Studies of Glx/Cr were largely negative with only one reporting a significantly higher Glx/Cr level in CRS than UTRS in putamen.

There were more significant findings when comparing UTRS with the FLR group including lower cortical thickness and brain volume of multiple brain regions as well as lowered Glx/Cr level in the dorsolateral prefrontal cortex. In fact, both treatment-resistant groups were found to have extensive differences in neurobiological features in comparisons with the FLR group. These suggested that the treatment-resistant patient groups have biologically distinct features compared with the FLR group and are likely to be a subtype of schizophrenia. Within the treatment-resistant patient group, the clozapine response group and clozapine-resistance group may share certain neurobiological features. However, a distinct hypo-frontality, abnormalities of frontal-striatal-basal ganglia circuitry as well as the GABA level differences may be neurobiological features differentiating the two treatment-resistant groups.

One of the strengths of this review study is covering results from multiple imaging modalities whilst focusing on specific regions of the brain to provide a comprehensive coverage of the neurobiological characteristics of UTRS. Secondly, the comparison was focusing specifically on differences between UTRS and CRS as well as FLR and HC and only studies with clearly stated operational definitions of different treatment outcome groups of schizophrenia were included. However, the limited sample size, diverse sample definitions and imaging modalities of existing studies make the conclusions difficult. Furthermore, heterogeneity of the TRS has been reported before, some developed TRS during the first episode and others after multiple relapses^5,60^, thus presence of multiple neurobiological characteristics of TRS and UTRS are possible. Various symptom mixtures of TRS patients might have also contributed to the diversity of results. Therefore, the conclusions with the current literature are far from complete in the pursuit of the understanding of neurobiological nature of different TRS groups and mechanisms of the TRS development. Only five studies adopted a longitudinal design, limiting examination of the effects of clozapine. On the other hand, clinical study of treatment-resistant populations with large sample sizes is challenging. Future multi-center collaborative studies are needed to examine neurobiological markers of clozapine-resistant schizophrenia using the current consensus definition of patient samples^6^, multimodal imaging tools and a longitudinal study design. Studying neurobiological changes because of the neuromodulation interventions of TRS could also be a viable strategy. Moreover, studies with better symptom characteristics of UTRS and focusing on specific symptom dimensions such as hallucination might better inform the neurobiological mechanisms of TRS and UTRS.

## Conclusions

This systematic review of neuroimaging studies comparing clozapine-resistant schizophrenia patients with patients responding to clozapine and patients responding to other antipsychotic medications found 17 studies with variable definitions of patient samples and study methodologies. The most consistent finding was the hypo-frontality of the clozapine-resistant group compared with the clozapine responsive group with a possible difference of frontal-striatal-basal ganglia circuitry as well as the GABA level between the two treatment-resistant patient groups. Extensive neurobiological differences were seen between the two treatment-resistant patient groups and patients responding to other antipsychotics. These suggest the treatment-resistant schizophrenia is likely to be a neurobiological subtype of schizophrenia. Clozapine-resistant and clozapine-response schizophrenia are likely to have some shared neurobiological features, but possible distinct features in the frontal lobe, frontal-striatal-basal ganglia circuitry as well as the GABA level. However, available studies are limited and define the need for multi-center collaborative studies using a consensus definition of patient samples, multimodal imaging tools, and longitudinal study designs.

### Supplementary information

Supplementary table
