# Supplementary material for: A systematic review of neuroimaging studies of clozapine-resistant schizophrenia
Source: Schizophrenia (Heidelb). 2023 Sep 26;9(1):65. doi: 10.1038/s41537-023-00392-7 (PMC10522657; doi:10.1038/s41537-023-00392-7)
Supplement: Supplementary file 1 — Supplementary table [file 41537_2023_392_MOESM1_ESM.docx]

**Supplementary Tables**

| Supplement Table 1 JBI critical appraisal Risk of Bias for Cross-sectional Studies | | | | | | | | |
| --- | --- | --- | --- | --- | --- | --- | --- | --- |
| Studies | Q1. Were the criteria for inclusion in the sample clearly defined? | Q2. Were the study subjects and the setting described in detail? | Q3. Was the exposure measured in a valid and reliable way? | Q4. Were objective, standard criteria used for measurement of the condition? | Q5. Were confounding factors identified? | Q6. Were strategies to deal with confounding factors stated? | Q7. Were the outcomes measured in a valid and reliable way? | Q8. Was appropriate statistical analysis used? |
| Ochi et al. 2022 | Y | Y | Y | Y | Y | Y | Y | Y |
| Ueno et al. 2022 | Y | Y | Y | Y | Y | Y | Y | Y |
| Iwata et al. 2021 | Y | Y | Y | Y | Y | Y | Y | Y |
| Kim et al. 2020 | Y | Y | UN | Y | Y | Y | Y | Y |
| McNabb et al. 2020 | Y | Y | Y | Y | Y | Y | Y | Y |
| Shah et al. 2020 | Y | Y | Y | Y | Y | Y | Y | Y |
| Tronchin et al. 2020 | Y | Y | UN | Y | Y | Y | UN | Y |
| Iwata et al. 2019 | Y | Y | UN | Y | Y | Y | UN | Y |
| McNabb et al. 2018 | Y | Y | UN | Y | Y | Y | UN | Y |
| Ahmed et al. 2015 | Y | Y | Y | Y | Y | Y | UN | Y |
| Anderson et al. 2015 | Y | Y | UN | Y | Y | Y | UN | Y |
| Goldstein et al. 2015 | Y | Y | UN | Y | Y | Y | Y | Y |
| Ertugrul et al. 2009 | Y | Y | UN | Y | Y | Y | Y | UN |
| Konicki et al. 2001 | Y | Y | Y | Y | UN | UN | Y | Y |
| Scheepers et al. 2001 | Y | Y | Y | Y | UN | Y | Y | Y |
| Rodríguez et al. 1997 | Y | Y | Y | Y | UN | UN | Y | Y |
| Rodríguez et al. 1996 | Y | Y | Y | Y | UN | UN | Y | Y |
| Honer et al. 1995 | Y | Y | Y | Y | Y | Y | Y | Y |
| Friedman et al. 1991 | Y | Y | Y | Y | Y | Y | Y | UN |
| Y, Yes; UN, unclear; N/A, Not applicable | | | | | | | | |

| Supplement Table 2 JBI critical appraisal Risk of Bias for Cohort Studies | | | | | | | | | | | |
| --- | --- | --- | --- | --- | --- | --- | --- | --- | --- | --- | --- |
| Studies | Q1. Were the two groups similar and recruited from the same population? | Q2. Were the exposures measured similarly to assign people to both exposed and unexposed groups? | Q3. Was the exposure measured in a valid, reliable way? | Q4. Were confounding factors identified? | Q5. Were strategies to deal with confounding factors stated? | Q6. Were the groups/participants free of the outcome at the start of the study (or at the moment of exposure)? | Q7. Were the outcomes measured in a valid and reliable way? | Q8. Was the follow-up time reported and sufficient to be long enough for outcomes to occur? | Q9. Was follow-up complete, and if not, were the reasons to loss to follow-up described and explored? | Q10. Were strategies to address incomplete follow-up utilized? | Q11.  Was appropriate statistical analysis used? |
| Tronchin et al. 2020 | Y | Y | UN | Y | Y | Y | UN | Y | UN | UN | Y |
| Ahmed et al. 2015 | Y | Y | Y | Y | Y | Y | UN | Y | UN | UN | Y |
| Ertugrul et al. 2009 | Y | Y | UN | Y | Y | Y | Y | UN | Y | Y | Y |
| Scheepers et al. 2001 | Y | Y | Y | Y | UN | Y | Y | Y | Y | Y | Y |
| Rodríguez et al. 1997 | Y | Y | Y | UN | UN | Y | Y | Y | Y | UN | Y |
| Rodríguez et al. 1996 | Y | Y | Y | UN | UN | Y | Y | Y | Y | UN | Y |
| Friedman et al 1991 | Y | Y | Y | Y | Y | Y | Y | UN | Y | UN | Y |
| Y, Yes; UN, unclear; N/A, Not applicable | | | | | | | | | | | |
